# Letter to the editor ‘Some optimization suggestions for “Impact of immediate postrecanalization cooling on outcome in acute ischemic stroke patients with a large ischemic core: prospective cohort study”’

**DOI:** 10.1097/JS9.0000000000001738

**Published:** 2024-05-29

**Authors:** Jiaan Lu, Hao Chi, Wei Fan, Juyi Wan

**Affiliations:** a School of Clinical Medicine, The Affiliated Hospital, Southwest Medical University; b Department of Cardiovascular Surgery, The Affiliated Hospital, Southwest Medical University, Metabolic Vascular Diseases Key Laboratory of Sichuan Province, Key Laboratory of Cardiovascular Remodeling and Dysfunction; c Key Laboratory of Medical Electrophysiology, Ministry of Education and Medical Electrophysiological Key Laboratory of Sichuan Province (Collaborative Innovation Center for Prevention of Cardiovascular Diseases), Institute of Cardiovascular Research, Southwest Medical University, Luzhou, Sichuan, People’s Republic of China

*Dear Editor,*

I am writing to express my gratitude for the insightful research conducted by Bai *et al*.^1^ titled ‘Impact of immediate postrecanalization cooling on outcome in acute ischemic stroke patients with a large ischemic core: prospective cohort study’. This survey delves into the emerging field of adjunctive therapy post-thrombectomy, aiming to improve the prognosis of patients with large-area ischemic stroke.

The design of this study deserves praise, as it includes the careful selection of patients and meticulous data collection. The use of a prospective cohort method, combined with the practical application of target temperature management (TTM), greatly increases the robustness of the research results. The inclusion of patients with large infarctions defined as ASPECTS ≤5 or an ischemic core ≥50 ml ensures that the results are clinically significant.

These results, although preliminary, indicate that TTM post-reperfusion may have neuroprotective advantages and increase the possibility of achieving favorable outcomes. The proportion of favorable outcomes in the TTM group is numerically higher, although not statistically significant. This finding warrants further investigation in larger-scale randomized controlled trials.

However, this study is not without limitations.

1. The sample size is moderate, which may limit the generalizability of the research results. Introducing a larger sample size can increase the applicability of the research findings^2^.
2. The baseline characteristics of the research group are not fully balanced, which may introduce confounding variables. For example, there is a higher proportion of males and smokers in the TTM group. Although efforts have been made to account for these imbalances in the analysis, the possibility of residual confounding cannot be completely ruled out. Methods to assess confounding variables or optimize statistical algorithms need to be introduced, such as subclassification, regression modeling, and the use of propensity scores for confounding assessment and adjustment in clinical trials^3^.
3. The external validity of this study may be limited because the results may not be fully applicable to a wider population or different treatment regimens^1^.
4. In addition to TTM, the article can discuss other possible treatment strategies, their advantages and disadvantages compared to TTM, and even involve a control group of another treatment strategy at the experimental design stage to assess the effects of different treatment strategies simultaneously^4^.

Given these considerations, it is necessary to cautiously interpret the results of this study. The potential benefits of TTM must be weighed against the risks of adverse events, such as pneumonia and deep vein thrombosis. As the incidence of adverse events is higher in the TTM group numerically, the article can delve into a more detailed analysis and discussion of the complications and side effects occurring in the TTM treatment group in order to better assess the risks and benefits of the treatment^1^. Furthermore, a deeper analysis and discussion of the differences in complications between the treatment group and the control group can be conducted. While the article briefly mentions the directions needed for future research, we believe that clearer directions and focuses for future research can be outlined to provide guidance for further studies in this field. For instance, the article can propose how to quantify the optimal approach to TTM treatment and indicate the need for future dose–response experiments to explore conclusions that are clinically feasible and universally applicable^5^.

The results of the study also underscore the need for carefully designed randomized trials to validate the effectiveness and safety of post-reperfusion cooling in improving the prognosis of patients with large ischemic strokes.

In summary, the study by Bai *et al*.^1^ provides valuable insights into the potential utility of TTM in the context of large ischemic strokes. The results are intriguing and warrant further exploration. Addressing the limitations of this study through rigorous, large-scale randomized trials is crucial for confirming the clinical utility of post-reperfusion cooling.

We appreciate the authors’ significant contributions to the field of stroke medicine, and we believe that the article lays the groundwork for advancements in this area. We eagerly anticipate future follow-up studies and improvements in experimentation in this field.

## Ethical approval

Not applicable.

## Consent

Not applicable.

## Sources of funding

This work was financially supported by the National Natural Science Foundation of China (82170325) and the Science and Technology Program of Sichuan Province (2022YFS0610).

## Author contribution

J.L., H.C., W.F., and J.W.: conceptualization, methodology, validation, formal analysis, investigation, resources, data curation, writing – original draft, writing – review and editing, supervision, and project administration.

## Conflicts of interest disclosure

The authors declares no conflicts of interest.

## Research registration unique identifying number (UIN)

Not applicable.

## Guarantor

Jiaan Lu, Hao Chi, Wei Fan, and Juyi Wan.

## Data availability statement

Not applicable.

## Provenance and peer review

Not applicable.
